# Elastin-Like Recombinamer Hydrogels for Improved Skeletal Muscle Healing Through Modulation of Macrophage Polarization

**DOI:** 10.3389/fbioe.2020.00413

**Published:** 2020-05-14

**Authors:** Arturo Ibáñez-Fonseca, Silvia Santiago Maniega, Darya Gorbenko del Blanco, Benedicta Catalán Bernardos, Aurelio Vega Castrillo, Ángel José Álvarez Barcia, Matilde Alonso, Héctor J. Aguado, José Carlos Rodríguez-Cabello

**Affiliations:** ^1^BIOFORGE (Group for Advanced Materials and Nanobiotechnology), CIBER-BBN, University of Valladolid, Valladolid, Spain; ^2^Servicio de Traumatología, Hospital Clínico de Valladolid, Valladolid, Spain; ^3^Servicio de Neurofisiología, Hospital Clínico de Valladolid, Valladolid, Spain; ^4^Servicio de Investigación y Bienestar Animal, University of Valladolid, Valladolid, Spain

**Keywords:** skeletal muscle healing, volumetric muscle loss, biomaterials, elastin-like recombinamers, hydrogels, macrophage polarization, immunomodulation

## Abstract

Large skeletal muscle injuries, such as a volumetric muscle loss (VML), often result in an incomplete regeneration due to the formation of a non-contractile fibrotic scar tissue. This is, in part, due to the outbreak of an inflammatory response, which is not resolved over time, meaning that type-1 macrophages (M1, pro-inflammatory) involved in the initial stages of the process are not replaced by pro-regenerative type-2 macrophages (M2). Therefore, biomaterials that promote the shift from M1 to M2 are needed to achieve optimal regeneration in VML injuries. In this work, we used elastin-like recombinamers (ELRs) as biomaterials for the formation of non- (physical) and covalently (chemical) crosslinked bioactive and biodegradable hydrogels to fill the VML created in the tibialis anterior (TA) muscles of rats. These hydrogels promoted a higher infiltration of M2 within the site of injury in comparison to the non-treated control after 2 weeks (*p*<0.0001), indicating that the inflammatory response resolves faster in the presence of both types of ELR-based hydrogels. Moreover, there were not significant differences in the amount of collagen deposition between the samples treated with the chemical ELR hydrogel at 2 and 5 weeks, and this same result was found upon comparison of these samples with healthy tissue after 5 weeks, which implies that this treatment prevents fibrosis. The macrophage modulation also translated into the formation of myofibers that were morphologically more similar to those present in healthy muscle. Altogether, these results highlight that ELR hydrogels provide a friendly niche for infiltrating cells that biodegrades over time, leaving space to new muscle tissue. In addition, they orchestrate the shift of macrophage population toward M2, which resulted in the prevention of fibrosis in the case of the chemical hydrogel treatment and in a more healthy-like myofiber phenotype for both types of hydrogels. Further studies should focus in the assessment of the regeneration of skeletal muscle in larger animal models, where a more critical defect can be created and additional methods can be used to evaluate the functional recovery of skeletal muscle.

## Introduction

Large skeletal muscle injuries are the result of high energy traumatisms as a consequence of different events, such as car accidents or explosions, being very common in clinics (Zalavras and Patzakis, 2003; Corona et al., 2015). They usually involve a volumetric muscle loss (VML), which implies an impairment of muscle function, and their treatment is a challenge for the orthopedic surgeon (Grogan et al., 2011; Greising et al., 2018). Currently, the gold standard is scar tissue debridement or autologous muscle transfer, which substantially increases donor site morbidity and can cause severe problems, such as infections or non-functional transfers (Lin et al., 2004, 2007; Klinkenberg et al., 2013).

After acute injuries, like VML, a complex process is activated in order to restore muscle structure and function, mainly due to the activation, proliferation and differentiation of a quiescent population of resident muscle progenitor stem cells known as satellite cells (SCs) (Tedesco et al., 2010; Lepper et al., 2011). These steps are orchestrated by the inflammatory response: during the first hours post-damage, circulating monocytes start differentiating into pro-inflammatory type-1 macrophages (M1) that activate the proliferation of SCs from the surrounding tissue (Tidball and Villalta, 2010; Saclier et al., 2013). Then, as the muscle repair process advances, the phenotype of the macrophages shifts to anti-inflammatory type-2 macrophages (M2), which express and secrete cytokines that stimulate myogenic differentiation of the SCs toward myofibers, thus being essential for a successful healing (Tidball and Villalta, 2010; Saclier et al., 2013). A dysregulated macrophage response leads to a chronic inflammation that induces the formation of a non-contractile fibrotic tissue due to the activation and recruiting of fibroblasts that secrete extracellular matrix (ECM) components, mainly collagen, to fill the void generated by the VML before it can be repopulated by new myofibers, hence leading to functional deficits (Järvinen et al., 2005; Shin et al., 2014).

During the past few years, several strategies have been proposed to modulate the inflammatory response to enhance skeletal muscle repair, many of them involving the use of biological (mainly decellularized porcine ECM) (Greising et al., 2017) or biomaterial-based scaffolds (Grasman et al., 2015). Interestingly, some of them have shown to promote a M2-balanced response (Sicari et al., 2014; Boersema et al., 2016; Wang et al., 2019). These scaffolds are intended to give mechanical and biochemical support to the different types of cells involved in the regeneration process, such as SCs, and they need to be biodegradable to give space for the formation of new myofibers (Wolf et al., 2015; Bartolacci et al., 2019). One specific type of scaffolds are injectable hydrogels, which are made up of polymeric biomaterials that form 3D networks with high water content and permeability and that can be applied in a minimally invasive way. While some of them are made of natural biomaterials, e.g., alginate or chitosan, meaning that they are extracted from natural sources, some others are made of synthetic ones (Qazi et al., 2015; Wolf et al., 2015). These synthetic biomaterials offer a better control on the chemical composition, and hence a high reproducibility, although they usually lack biological activity (O’Brien, 2011).

Within the different types of synthetic biomaterials, we can identify recombinant polymers (recombinantly expressed structural proteins with repetitive domains) (Cappello et al., 1990; Tirrell et al., 1991), such as elastin-like recombinamers (ELRs) (Rodríguez-Cabello et al., 2009). These molecules derive from the repetition of the L-Val-L-Pro-Gly-X-Gly (VPGXG) pentapeptide found in natural elastin, where X can be any amino acid except L-Pro, and are able to self-assemble through hydrophobic interactions above the so-called transition temperature (*T*_*t*_) (Urry et al., 1976; Urry, 2006; Ibáñez-Fonseca et al., 2019). Due to their recombinant nature, they can be precisely engineered at the DNA level to bear specific amino acids (Rodríguez-Cabello et al., 2009; Girotti et al., 2015). For instance, the introduction of lysines with amine groups that can be modified for covalent crosslinking through “click chemistry” strategies, like strain-promoted alkyne-azide cycloaddition (SPAAC), may allow the formation of chemical hydrogels (González de Torre et al., 2014; Madl et al., 2016). On the other hand, physical hydrogels can be achieved by the inclusion of amino acid sequences able to form stable non-covalent interactions (e.g., H-bonds), such as the repetitive domains found in silk fibroin from *Bombyx mori* silkworm that, in combination with the elastin-like building blocks, form hydrogels through a concomitant self-assembly above the *T*_*t*_ (Fernández-Colino et al., 2014; Ibáñez-Fonseca et al., et al., 2020). Furthermore, the genetic fusion of bioactive sequences, including cell adhesion domains, like the L-Arg-Gly-L-Asp (RGD) tripeptide (Ruoslahti, 1996), or protease-sensitive sequences for improved biodegradation (Flora et al., 2019; Contessotto et al., under review), permits the obtaining of hydrogels with acquired functionalities. In this last regard, the inclusion of motifs sensitive to matrix metalloproteinase (MMP)-2, 9 and 13 provides a multipurpose platform able to be degraded in different *in vivo* environments (Lutolf et al., 2003; Chung et al., 2006; Contessotto et al., under review). Therefore, due to their intrinsic properties of high biocompatibility (Ibáñez-Fonseca et al., 2018), mechanical stability (Fernández-Colino et al., 2014; González de Torre et al., 2014), injectability (Martín et al., 2010; Fernández-Colino et al., 2014), and acquired bioactivity (Girotti et al., 2004; Ibáñez-Fonseca et al., 2017), ELR-based hydrogels have found several uses in tissue engineering and regenerative medicine (Coletta et al., 2017; Pescador et al., 2017; Staubli et al., 2017; Contessotto et al., under review), specially within the field of *in situ* tissue regeneration (Lee et al., 2016).

In this work, we propose the use of chemical and physical ELR-based hydrogels, both of them biodegradable, to improve the healing of skeletal muscle injuries. Our hypothesis is that ELR hydrogels will be able to modulate the macrophage response and facilitate the shift to pro-regenerative M2 macrophages. Moreover, the ELR hydrogels will provide a cell-friendly and biodegradable environment that will prevent the formation of fibrotic tissue in the area of the defect, and that will allow the development of new myofibers. Therefore, the objective of this study was to quantitatively analyze macrophage polarization and its effects on muscle healing, in terms of collagen deposition (fibrosis) and muscle morphology, following ELR hydrogel treatment in a rat model of VML.

## Materials and Methods

### ELRs Biosynthesis and Characterization

The ELRs used in this work were biosynthesized through recombinant DNA technology as described elsewhere (Rodríguez-Cabello et al., 2012). Briefly, the genes encoding for the recombinamers were cloned into a pET-25b(+) plasmid vector (Novagen, Merck, Germany) that was used to transform a BLR(DE3) strain of *Escherichia coli* (Novagen, Merck, Germany). An ELR-expressing clone was cultured in a 15-L bioreactor (Applikon Biotechnology B.V., Netherlands) and the ELR was purified by several cooling and heating cycles with centrifugation steps. Then, the highly pure ELR solution was dialyzed against ultra-pure water and filtered through 0.22 μm filters (Nalgene, Thermo Fisher Scientific, United States) for sterilization. Finally, the solution was freeze-dried prior to storage.

Two of the ELRs used in this work, namely HRGD6 and HE5, i.e., the ones used for the formation of chemically crosslinked hydrogels (or simply chemical hydrogels), were previously described (Costa et al., 2009; Contessotto et al., under review). HRGD6 has six cell adhesion RGD sequences per molecule, embedded within the lysine-containing elastin-like backbone, whereas the HE5 includes MMP-sensitive domains for biodegradation and lysine-rich crosslinking domains within a glutamic acid-containing elastin-like backbone. The presence of lysines in both ELRs makes them suitable for chemical modification and subsequent covalent crosslinking via “click chemistry” for the formation of chemical hydrogels (see below).

On the other hand, the silk-elastin-like recombinamer (SELR) used for the formation of physically crosslinked hydrogels, the so-called IKRS-MMP, was based on a previously designed SELR (Ibáñez-Fonseca et al., 2020), to which a MMP-sensitive domain, similar to the one included in the HE5, was included for biodegradation.

The characterization methods for every ELR batch included sodium dodecyl sulfate polyacrylamide gel electrophoresis (SDS-PAGE) and matrix-assisted laser desorption/ionization time-of-flight (MALDI-TOF) for the evaluation of the purity and the molecular weight, HPLC to determine the amino acid composition and differential scanning calorimetry (DSC) for the calculation of the transition temperature. Furthermore, the endotoxin levels were assessed by the limulus amebocyte lysate assay with the Endosafe^®^-PTS system (Charles River Laboratories, Inc., United States) and were always below 1 endotoxin unit/mg of ELR.

### ELR Chemical Modification

The chemical modification of the ELRs was performed as previously described (González de Torre et al., 2014). On one hand, the ELR containing RGD cell-adhesion domains (HRGD6) was chemically modified with azide groups through the transformation of the ε-amine group found in the side chain of lysine residues, achieving a 55–65% of modification (from 14 to 16 modified lysines, out of 24), and giving a HRGD6-N_3_. On the other hand, the ELR comprising MMP-sensitive motifs (HE5) was modified similarly, in this case to bear cyclooctyne (activated alkyne) groups, resulting in a 30–40% of modification (from 3 to 4 modified lysines, out of 9), and named HE5-C. These modified ELRs were used for the formation of covalently crosslinked “click” ELR-based hydrogels (chemical hydrogels).

### ELR-Based Hydrogel Preparation and *in vivo* Administration

In this work, two different types of biodegradable hydrogels were used to evaluate their influence on muscle healing: non-covalently crosslinked SELR-based hydrogels (physical hydrogels) and covalently crosslinked “click” ELR-based hydrogels (chemical hydrogels), formed through SPAAC (González de Torre et al., 2014). In both cases, the recombinamers were dissolved in cold 1× PBS for 16–24 h at 4°C and the hydrogels were formed *in situ* just after the creation of the muscle defect.

In the case of the “click” ELRs, the HRGD6-N_3_ and the HE5-C were dissolved separately and mixed prior to injection in a 1:1.8 ratio, since this was found to be the optimal proportion, taking into account the different molecular weights and modification percentage of the ELRs, finally giving 50 mg/mL hydrogels. The mixture was left for 8 min in an ice bath and afterward it was placed in the injury site with a pipette, where the hydrogel formation process was completed.

For the physical hydrogel (IKRS-MMP), the mono-component solution was left in an ice bath until its administration in the site of the defect with a pipette, similarly to the chemical hydrogel. In this case, the gelation was triggered by the change in the temperature of the solution that leads to an inverse temperature transition (ITT) and to the formation of a network through hydrophobic interactions. The hydrogel was further stabilized by the folding of silk domains into β-sheets, which results in crystallization (Fernández-Colino et al., 2014; Ibáñez-Fonseca et al., 2020).

In both cases, administration was easily performed with a pipette, taking advantage of the injectability of both hydrogels, and the gelation was instantaneous, which avoided the dilution of the hydrogel once implanted. Moreover, due to the inclusion of a MMP-sensitive amino acid sequence in the HE5 and IKRS-MMP, both types of hydrogels, i.e., chemical and physical, were biodegradable.

### Animal Experiments

#### Ethical Statement

All animal experiments were conducted in accordance with the institutional guidelines for the care and use of experimental animals of the University of Valladolid (Spain) in accordance with Directive 2010/63/EU. The protocol was approved by the Committee of Ethics in Animal Experimentation and Welfare (CEEBA, for its Spanish acronym) of the University of Valladolid (protocol number 5402485).

#### Animal Care

A total of 19 three-month-old male Wistar rats were used in this study. The average weight was 400 g at the time of surgery. The animals were kept in cages with a light:dark cycle of 12:12 and provided with *ad libitum* food and water. An identification chip was placed in the interscapular region, inaccessible to the animals, to ensure masking during the study.

#### Experimental Groups

Four different groups were established through randomized classification: non-treated or empty (*n* = 11), treated with chemical biodegradable hydrogels (*n* = 11) or physical biodegradable hydrogels (*n* = 11), and non-injury or healthy group (*n* = 5). In the empty group, the VML was left untreated, whereas for the treated groups chemical and physical biodegradable hydrogels were placed in the injury area. No surgical procedure was done in the non-injury group.

#### Anesthesia

All surgical procedures were carried out under proper anesthesia. Intraperitoneal anesthesia of the animals was performed with ketamine-medetomidine at a dose of 0.125 mL per 100 g of animal weight.

#### Tibialis Anterior VML Injury

Our goal was to create a defect of at least a 20% of the weight of the tibialis anterior (TA) muscle, which was calculated according to the equation described by Wu et al. (2012), giving an average defect weight of 121 mg. A scheme of the TA VML injury has been included in Supplementary Figure 1A.

First, the inferior limbs of the animal were shaved using an electric shaver to facilitate subsequent procedures. Then, the animal was placed in the supine position on a heat blanket (to prevent hypothermia), and it was covered with a sterile drape, so that only the legs were accessible. Subsequently, a longitudinal incision was made in the skin from the knee to the ankle following the course of the TA muscle with a sterile no. 11 blade scalpel. The fascia was sectioned independently and separated from the muscle using blunt dissection to completely uncover the anterior surface of the TA.

Afterward, a transversal mark was made in the exposed muscle with a sterile marker, measuring 1cm from the tibial tuberosity with a rule. This mark would be the proximal limit of the defect. A second mark was made parallel to the first, 1 cm from it. In this way, the proximal and distal limits of the defect were delimited. Regarding the width of the VML, we left a margin of about 2 mm from the medial and lateral margins of the TA muscle. In this way, both the length and width of the defect were precisely delimited, whereas the depth was adjusted according to the volume of muscle needed to achieve the above calculated weight.

Once the defect was created, the corresponding treatment was applied, according to the groups defined above. For the administration of the hydrogel, the skin was first sutured with Vicryl rapid 2/0, and the skin was left partially open during the operation to minimize mobilization of the hydrogel during closure. Both chemical and physical hydrogels were formed instantaneously once implanted, as previously described in the hydrogel preparation section. Subsequently, the continuous suture was tensed and knotted. A self-adhesive bandage of both legs was made so that the rats could not contaminate or bite the wound.

Animals were provided with postoperative analgesia in food and drink. Specifically, ibuprofen was dissolved in water at a concentration of 10 mL/L of water for 3 days, and tramadol was administered masked in commercial hazelnut cocoa spread at a concentration of 1 mg/kg/day, calculated for 48 h.

#### Euthanasia

Animals were euthanized 2 and 5 weeks post-injury by intracardiac injection of phenobarbital, after being anesthetized to avoid suffering. Then, whole TA muscles were extracted for processing and analysis as described below.

### Histological Processing

The TA muscles were harvested and divided into two halves in the middle area of the defect, and they were used to achieve both longitudinal and cross-sections (see Supplementary Figure 1B for a schematic representation). To this end, the samples were mounted on cork discs according to the direction of the muscle fibers using a small amount of optimum cutting temperature (OCT) mounting medium (VWR, United States). Then, the samples were frozen in 2-Methylbutane (Thermo Fisher Scientific, Belgium) previously chilled in liquid nitrogen. After freezing, the samples were stored at −80°C until further processing. Subsequently, 6 μm cross-section slices were cut in a cryostat (Thermo Fisher Scientific, United States) and stained with Harris’ hematoxylin (Merck, Germany)/eosin-Y (Sigma-Aldrich, United States) (HE staining) and Picrosirius red (Abcam, United Kingdom) following the manufacturers’ protocols.

### Histomorphometry

Histomorphometry methods were used to quantify areas, number and size of myofibers, and collagen percentage. For this purpose, HE images of the whole muscle sections were obtained using a Nikon Eclipse 80i microscope (Nikon Corporation, Japan) coupled to an automated stage (Prior, United Kingdom) and a DS-Fi1 camera (Nikon Corporation, Japan), which were controlled with the NIS-Elements AR software (Nikon Corporation, Japan).

**FIGURE 1:**
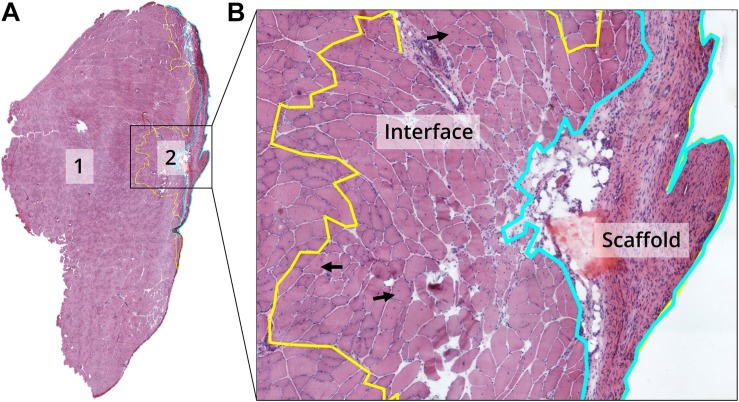
Example of TA muscle cross-section and visual explanation of the different areas used for the quantifications. **(A)** Example picture of the hematoxylin–eosin (HE) staining of the entire TA muscle (×20 magnification), where we identified and manually traced the remaining native tissue (1) and the injury area (2). **(B)** Visual explanation of the division of the injury area into two clearly differentiated regions: interface (showing newly formed myofibers with central nuclei, highlighted with black arrows) and scaffold (no visible myofibers are present). Therefore, both the scaffold and interface areas conform the potentially injured/wounded region.

Within the samples, three different regions were differentiated: the remaining native tissue, the interface (tissue newly regenerated between the remaining muscle and the scaffold area, characterized by the presence of myofibers with internal nuclei) and the scaffold area (without myofibers). The sum of these last two areas give the area corresponding to the potentially injured tissue, i.e., the region where the VML was created (Figure 1), although it may not represent the wound area to its full extent. The three different areas were traced manually and measured using the Fiji distribution of the ImageJ software (Schindelin et al., 2012). The percentage of injury (interface and scaffold) area was normalized to the entire muscle area, whereas the percentage of the interface and scaffold areas were obtained by normalizing to the injury area.

Automatic myofiber counting was performed using a customized macro that included the use of the Trainable Weka Segmentation tool in Fiji (ImageJ) (Arganda-Carreras et al., 2017). Myofiber size was determined by measuring the lesser diameter (minimal Feret’s, defined as the closest distance between the two parallel tangents of the muscle fiber) of 100 fibers manually in four different locations (Briguet et al., 2004; Dubach-Powell et al., 2008; Pertl et al., 2013) in the interface region of each sample (*n* = 5 per group/time point). Data were represented as frequency distribution of size ranges with Gaussian distribution with GraphPad Prism 6.0 software. The quantification of the percentage of myofibers with internal nuclei was performed similarly by counting the nuclei of a total of 100 fibers in four different locations within the interface region of each sample (*n* = 5 per group/time point).

The entire muscle section was analyzed for collagen staining with Picrosirius red under bright-field and polarized light. The birefringent staining under polarized light is highly specific for type I and III mature collagen fibrils (Junqueira et al., 1979), and it was used for collagen quantification with a custom macro in Fiji (ImageJ).

### Immunofluorescence Staining

Immunofluorescence stainigs were performed as previously described (Aurora et al., 2016) to detect macrophages: type-1 or pro-inflammatory macrophages (anti-CCR7 antibody, 1:200; ab32527, Abcam, United Kingdom) (Corona et al., 2013) and type-2 or anti-inflammatory macrophages (anti-mannose receptor (CD206) antibody, 1:200; ab64693, Abcam, United Kingdom) (Lankford et al., 2018). Alexa Fluor 488-labeled secondary antibody (1:500; ab150077, Abcam, United Kingdom) was used for final immunostaining in both cases. At least 16 non-overlapping images (20× magnification) of the injury region were randomly taken with a Nikon Eclipse Ti-E coupled to a Nikon DS-2MBWc digital camera (Nikon Corporation, Japan). The quantification of the number of cells in each image was obtained manually with Fiji (ImageJ).

### Statistical Analysis

All the results are presented as means ± SD (*n* = 5, unless otherwise stated in figure caption). *p*-values were calculated using the one-way (differences between more than 2 groups) or 2-way ANOVA (including time-dependence) with Tukey’s multiple comparisons test using GraphPad Prism 6.0 software. All *p*-values < 0.05 were considered significant. ^∗^*p* < 0.05, ^∗∗^*p* < 0.01, ^∗∗∗^*p* < 0.001, ^****^p < 0.0001, nsd, not significantly different.

## Results

The VML injury was successfully created in the rat TA muscle as described above, and muscle samples were harvested at 2 and 5 weeks post-injury. No post-implantation mortality was detected and no implant rejection was observed. No significant differences between groups were found as regards initial animal body weight, defect weight and percentage of excised TA muscle (Supplementary Table 1).

**FIGURE 2:**
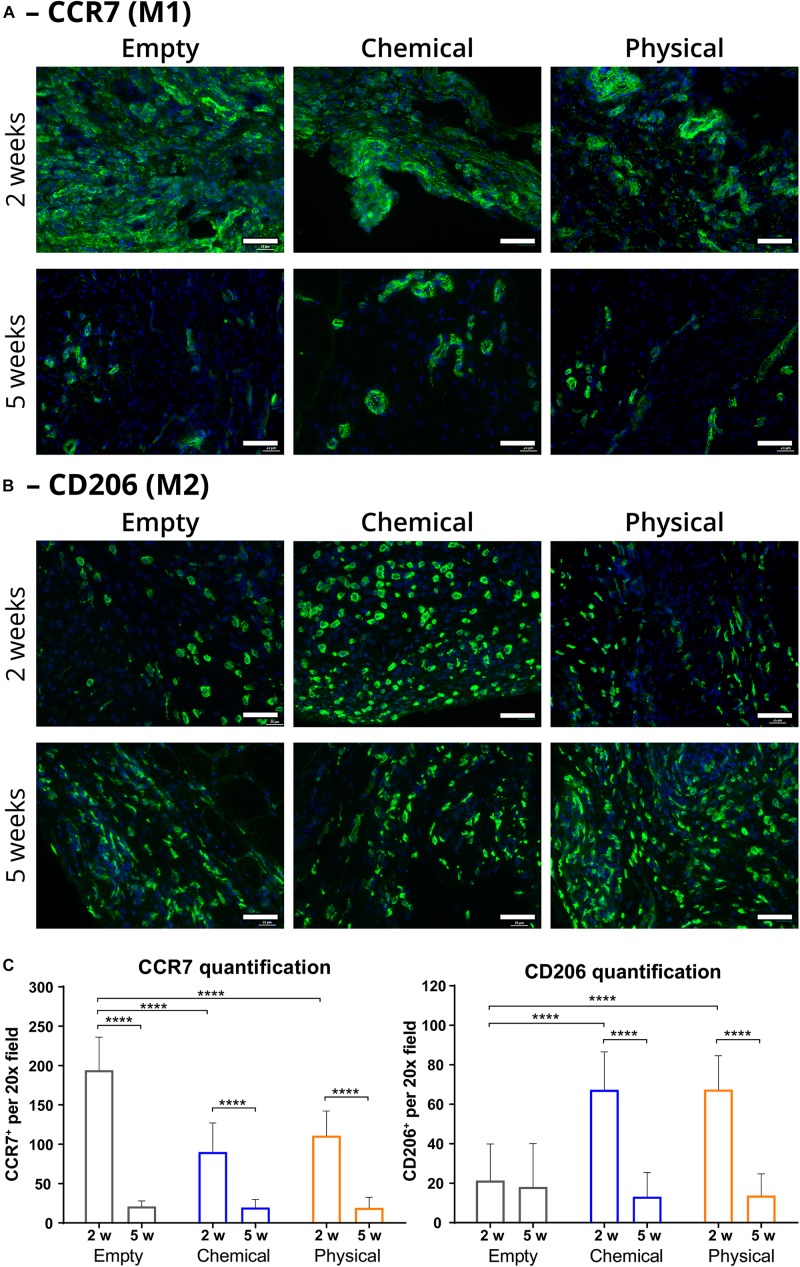
Immunofluorescence representative images and quantification of macrophage populations in the injury area (scaffold and interface regions). **(A)** Type-1 macrophages (M1 or pro-inflammatory; in green) present in the injury area of samples from the empty (left), chemical (center), and physical (right) groups after 2 (top) and 5 weeks (bottom), labeled with an anti-CCR7 antibody. **(B)** Type-2 macrophages (M2 or anti-inflammatory; in green) present in the injury area of samples from the empty (left), chemical (center), and physical (right) groups after 2 (top) and 5 weeks (bottom), labeled with an anti-CD206 antibody. **(C)** Quantification of macrophage populations in the injury area (number of CCR7- or CD206-positive cells per 20× field) at 2 (2 w) and 5 weeks (5 w) post-injury. All the samples were counterstained with DAPI for nuclei. Scale bar = 50 μm. *n* = at least 16, which are the number of 20× fields used for the quantification. ****p < 0.0001.

To assess our hypothesis, we first performed immunostaining toward M1 (CCR7^+^) and M2 (CD206^+^) macrophages (Figures 2A,B, respectively) and quantified the number of each type of immune cells, observing that the injury areas of the samples treated with either the chemical or the physical hydrogels showed a significantly greater quantity of M2 than the empty (non-treated) samples (*p* < 0.0001) at 2 weeks post-injury (Figure 2C). Moreover, the presence of M1 in the hydrogel-treated samples was significantly lower than in the empty samples at this timepoint (*p* < 0.0001), thus giving a much higher M2/M1 ratio for the hydrogel-treated samples. These ratios were 0.75 ± 0.38 and 0.61 ± 0.23 for the groups treated with the chemical and with the physical hydrogel, respectively, while it was 0.11 ± 0.10 for the empty group. On the other hand, the quantity of M1 and M2 macrophages decreased for every group after 5 weeks, giving similar values for all of them (nsd). Nevertheless, the difference in M2 for the empty group between 2 and 5 weeks was not significant, meaning that the quantity of this type of macrophages did not peak at 2 weeks, contrarily to what we observed for the hydrogel-treated samples.

**FIGURE 3:**
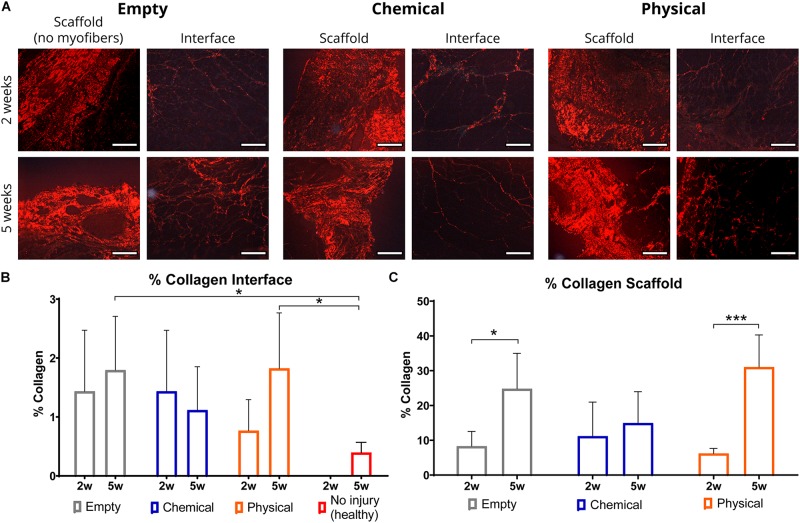
Representative images and quantification for collagen staining with Picrosirius red. **(A)** Polarized light images from muscle samples of empty (left), chemical (center), and physical (right) groups for the scaffold (area without newly formed myofibers) and interface (area with newly formed myofibers) regions at 2 (top) and 5 weeks (bottom) post-injury. Scale bar = 100 μm. **(B,C)** Percentage of collagen in the interface (including no injury group at 5 weeks) and scaffold regions, normalized to their areas, after 2 and 5 weeks. *p < 0.05, ***p < 0.001.

Since one of the main outcomes of a balanced macrophage response is the healing of the damaged tissue with a lesser amount of fibrosis, we performed Picrosirius red histological staining to observe collagen deposition in the muscle samples. For this purpose, we used polarized light (see Supplementary Figure 2 for the bright-field pictures) to specifically differentiate and quantify collagen within the muscle sections for comparison between groups (Figure 3A). In particular, there were not significant differences in collagen deposition in the interface area (injury region with newly formed myofibers) between 2 and 5 weeks post-injury, although in the empty and physical hydrogel groups, but not in the chemical hydrogel one, there was a tendency toward increasing levels along time (Figure 3B). On the other hand, there was a significant increase in collagen deposition in the scaffold area (injury region without newly formed myofibers) from 2 to 5 weeks for the empty and physical hydrogel groups (*p* < 0.05, respectively), whereas this was not observed for the chemical hydrogel-treated samples (Figure 3C). Moreover, the interface (remodeling) area of the samples treated with the chemical hydrogel after 5 weeks showed similar levels of collagen compared to the total percentage of collagen presented in the non-injured (healthy) samples (Figure 3B), while the empty and physical hydrogel groups presented significantly higher collagen levels (*p*<0.05).

**FIGURE 4:**
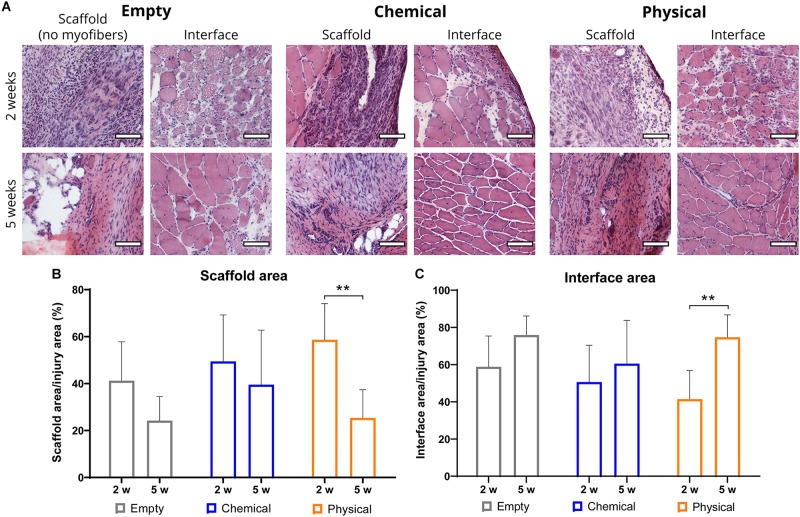
**(A)** Representative images of the hematoxylin–eosin (HE) staining of sample cross-sections of the scaffold (area without newly formed myofibers) and interface (area with newly formed myofibers) regions (left and right for each group, respectively) of samples from the empty (left), chemical (center), and physical (right) groups at 2 (top) and 5 weeks (bottom) post-injury. Scale bar = 100 μm. **(B,C)** Percentage of the interface and scaffold areas, with respect to the injury area at 2 and 5 weeks post-injury. **p < 0.01.

Finally, we also performed hematoxylin and eosin (HE) staining of the muscle cross-sections to observe their morphology (see Supplementary Figure 3 for the entire sections). In all the samples, we identified the remaining native tissue and the injury area, divided in the interface and the scaffold areas, as shown in Figure 1, which were subsequently quantified. Specifically, quantification of the interface and scaffold areas (Figure 4A) presented no significant differences between the different treatment groups (Figures 4B,C). Nevertheless, we observed significant differences in the muscles treated with the physical hydrogel between 2 and 5 weeks post-injury in both the interface and the scaffold areas (Figures 4B,C), suggesting a greater remodeling of the muscle tissue during this time. Moreover, we found an almost complete absence of myofibers with internal nuclei in the healthy tissue (Supplementary Figure 4), which suggests that the healing process only takes place in the interface and scaffold areas that represent the potentially injured region.

**FIGURE 5:**
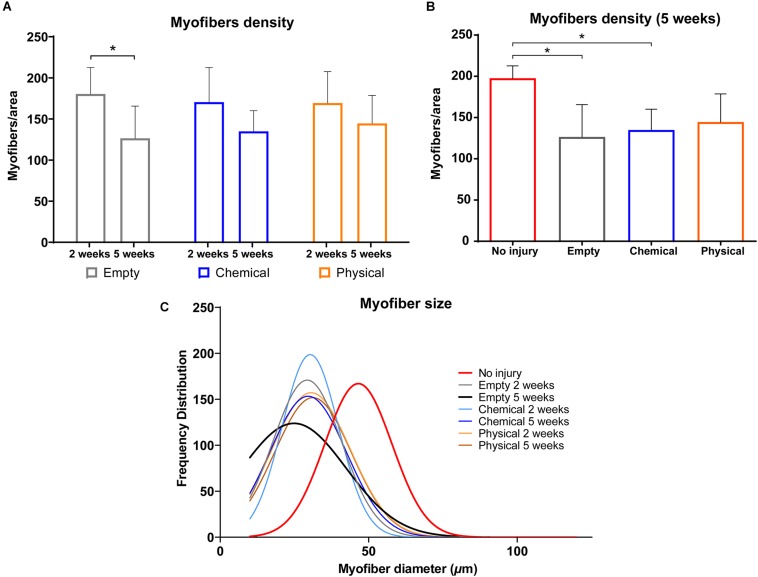
Histomorphometric characterization of the HE-stained samples. **(A)** Myofiber density at 2 and 5 weeks post-injury for the empty (gray), chemical (blue) and physical (orange) groups. **(B)** Myofiber density after 5 weeks for the empty (gray), chemical (blue) and physical (orange) groups in comparison with no injury (healthy) samples (red). **(C)** Myofiber size distribution (number of myofibers with a specific diameter) for the different groups used in this study, i.e., empty, chemical, and physical, after 2 and 5 weeks, in comparison with the no injury (healthy) group. *p < 0.05.

Further parameters, such as myofiber density, were also quantified. In this case, we found that the density in the interface area of samples from the empty group showed a significant decrease from 2 to 5 weeks (*p* < 0.05) (Figure 5A), which correlates to a lower myofiber number. On the other hand, no differences in the chemical and physical groups were observed. In addition, when samples harvested at 5 weeks from the three groups were compared to healthy samples (no injury), all of them showed a lower cell density, although in the groups treated with the physical hydrogel this difference was not significant (Figure 5B).

Another parameter that allowed the comparison between groups was the size of the myofibers, i.e., the cross-sectional minimal Feret’s, which permits a reliable measurement of myofiber diameter independently of variations in sample orientation (Pertl et al., 2013). These results showed that the size of the myofibers found in the hydrogel-treated samples were more similar to the size of the healthy ones than the cells in the empty samples at 5 weeks post-injury (Figure 5C), although all the injured groups presented smaller myofibers at 2 and 5 weeks than the uninjured samples.

## Discussion

The results obtained in this study show that the ELR hydrogels, both chemical and physical, promote a shift in macrophage polarization toward anti-inflammatory M2, highlighted by the higher M2/M1 ratio in comparison with the untreated control at 2 weeks post-injury. As aforementioned, M2 are known to secrete anti-inflammatory cytokines, while also regulating myogenic differentiation. Therefore, the increase in M2 for the hydrogel-treated samples at 2 weeks post-injury should promote a better regulation of the skeletal muscle repair in comparison with the untreated control, resulting in a morphology more similar to the healthy muscle and with a less amount of fibrotic/scar tissue. In this last regard, we studied collagen content by Picrosirius red staining, and it revealed that there was less collagen in the hydrogel-treated samples in comparison to the untreated control, especially in the case of the chemical hydrogel, which is indicative of a lesser fibrosis. Similarly, histological results through hematoxylin-eosin staining showed that the morphology of the muscle treated with the ELR hydrogels is more similar to the healthy one than the untreated control at 5 weeks post-injury, which is clearly evident when measuring specific parameters, such as myofiber density and diameter. This effect is consistent with the higher M2 response observed, which induces the maturation of the myofibers to give a more healthy-like muscle, while untreated samples, where the presence of M2 is much lower than in the treated samples at 2 weeks post-injury, show smaller myofibers, suggesting a lack of complete myogenic differentiation and muscle growth.

In this work, we show how the use of a bioactive and dynamic synthetic scaffold promotes an enhanced healing of injured skeletal muscle by itself, through the immunomodulation of the macrophage response that guides the repair process. The therapeutic strategy presented herein belongs to the field of *in situ* tissue regeneration, where a host cell recruitment is achieved (Lee et al., 2016), in this case without the use of external cells or growth factors, hence preventing potential side effects (Baldo, 2014). The immunomodulation shown by ELR hydrogels has also been observed in other works that used biological scaffolds, mainly decellularized urinary bladder matrix (UBM), to improve the healing of VML injuries. In some of them, authors do not observe a shift from a M1 to a M2 response, and, as they state, this leads to an impaired muscle healing (Aurora et al., 2015, 2016; Greising et al., 2017), whereas some others report dissimilar results (Sicari et al., 2014; Dziki et al., 2016; Lee et al., 2019).

On the other hand, it has been suggested that the treatment with synthetic biomaterial-based scaffolds, comparable to the ELR hydrogels used in this work, also promote a M2-balanced immune response, with different examples using a photoresponsive hyaluronan hydrogel (Wang et al., 2019), a biohybrid pNIPAAm and UBM hydrogel (Zhu et al., 2018), keratin (Passipieri et al., 2017), or fibrin by itself (Tanaka et al., 2019). This strategy has several advantages in comparison with biological scaffolds, since the controlled synthesis of the biomaterials used for their fabrication does not rely on methods like decelullarization.

Regarding the immunomodulation mediated by ELR hydrogels, we suggest that, on one hand, the presence of RGD promotes a physiological interaction with the scaffolds, that hence resemble some of the properties of the native ECM. Another reason that may provide an explanation to the findings of this work is the fact that the ELR hydrogels used herein are biodegradable. This means that they act as a transient ECM-like scaffold, providing a dynamic environment that evolves as required by the cells involved in the healing process, which secrete MMPs relevant for the proteolysis of the hydrogels [mainly macrophages (Turner and Badylak, 2012)] and make space for the formation of regenerated tissue. Indeed, we found in a preliminary study that biodegradable ELR hydrogels are completely necessary for the healing of the VML injury, which otherwise is permanently occupied by the scaffold (Supplementary Figure 5). Previous studies have shown the importance of the biodegradation of ELR hydrogels with potential application in tissue engineering, influencing, for instance, vascularization (Staubli et al., 2017; Flora et al., 2019), which is considered one of the main events to achieve a successful healing of damaged tissues. In addition, the filling of the void created through the VML with the ELR hydrogels impedes the formation of a large scar tissue (fibrosis) that usually impairs muscle healing (Järvinen et al., 2005; Turner and Badylak, 2012). Therefore, the use of biodegradable ELR hydrogels could provide a niche that promotes vascularization and replacement by newly formed skeletal muscle, resulting in an efficient healing.

This investigation has a main limitation, which is the lack of functional characterization of the TA muscles. Nevertheless, we aimed to delve into the effect of ELR hydrogels in the healing of VML injuries in terms of cell and molecular biology, in order to set the basis for future works with larger animal models (Pollot and Corona, 2016). These models will not only be more relevant as regards the future application of ELR hydrogels in the treatment of skeletal muscle injuries in humans, but they will also allow the use of non-invasive techniques already implemented in clinics for the determination of muscle function.

## Conclusion

In conclusion, this study demonstrates that bioactive and biodegradable ELR hydrogels regulate the macrophage response by inducing M2 polarization after a VML injury. This immunomodulation results in an enhanced skeletal muscle healing, with a reduced collagen deposition and a muscle morphology more similar to the healthy tissue. Therefore, we confirmed that ELR hydrogels provide a cell-friendly and dynamic environment that induces M2 shift and supports an enhanced healing, especially in the case of the chemically crosslinked ELR hydrogel, which showed a reduced fibrosis. The work presented here paves the way for future studies in more relevant large animal models of VML treated with the chemical ELR hydrogel that include functional characterization.

## Data Availability Statement

Data associated with this study is available upon request to the corresponding author.

## Ethics Statement

The animal study was reviewed and approved by the Comité de Ética en Experimentación y Bienestar Animal (CEEBA) de la Universidad de Valladolid (protocol number 5402485).

## Author Contributions

AI-F, SS, DG, MA, HA, and JR-C designed the study. AI-F, SS, DG, BC, ÁÁ, and HA performed the experiments. AI-F, SS, and DG wrote the manuscript. AV, MA, HA, and JR-C revised the manuscript. All authors approved the final version.

## Conflict of Interest

The authors declare that the research was conducted in the absence of any commercial or financial relationships that could be construed as a potential conflict of interest.
