# Supplementary material for: Elastin-Like Recombinamer Hydrogels for Improved Skeletal Muscle Healing Through Modulation of Macrophage Polarization
Source: Front Bioeng Biotechnol. 2020 May 14;8:413. doi: 10.3389/fbioe.2020.00413 (PMC7240013; doi:10.3389/fbioe.2020.00413)
Supplement: Supplementary file 1 [file Data_Sheet_1.PDF]

Supplementary Material

1 Supplementary Figures and Tables

1.1 Supplementary Tables

Table S1. Experimental data obtained as regards initial animal body weight, defect weight and percentage of TA muscle volume excised, according to Wu et al. (Wu et al., 2012), for the different groups at each time point. Values are represented as Mean ± SD. No significant differences were observed between groups for all the different parameters.

Experimental group	Time point	Initial body weight (g)	Defect weight (mg)	% of TA muscle volume excised
Chemical	2 weeks	353.7 ± 25.9	176.5 ± 22.8	33.4 ± 3.7
	5 weeks	365.8 ± 59.2	173.2 ± 25.6	32.4 ± 6.2
Physical	2 weeks	362.5 ± 64.7	173.2 ± 25.5	33.1 ± 9.7
	5 weeks	361.5 ± 44.1	165.3 ± 18.0	31.4 ± 8.7
Empty (untreated)	2 weeks	371.7 ± 62.2	159.2 ± 22.2	28.9 ± 4.5
	5 weeks	374.8 ± 43.7	178.0 ± 23.1	32.2 ± 7.8

1.2 Supplementary Figures

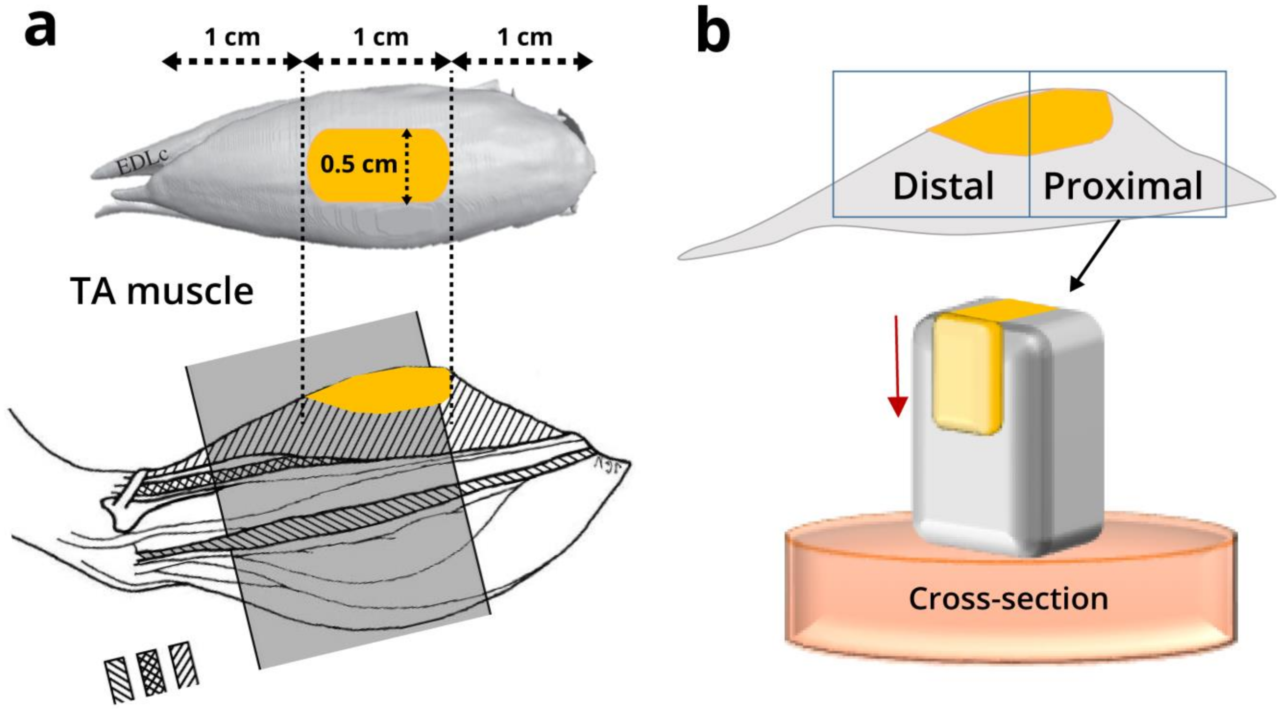

Supplementary Figure 1. (a) Schematic anatomic representation of the VML injury in the rat TA muscle, as it was surgically created. (b) Schematic representation of sample collection and orientation prior to freezing for histological processing.

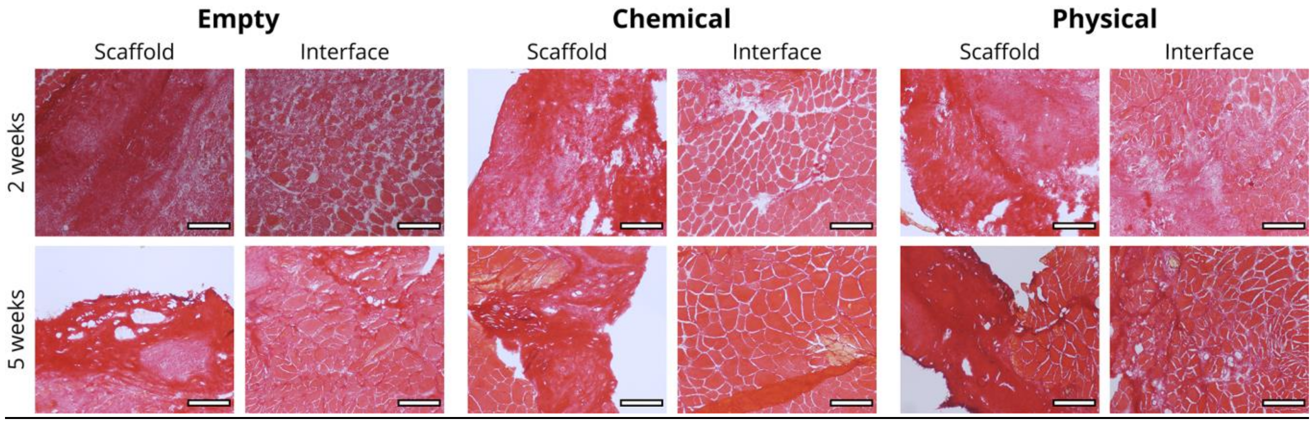

Supplementary Figure 2. Brightfield images of the Picrosirius Red staining of sample cross-sections of the scaffold and interface regions (left and right for each group, respectively) of samples from the empty (left), chemical (centre) and physical (right) groups at 2 (top) and 5 weeks (bottom) post-injury. Scale bar = 100 μm.

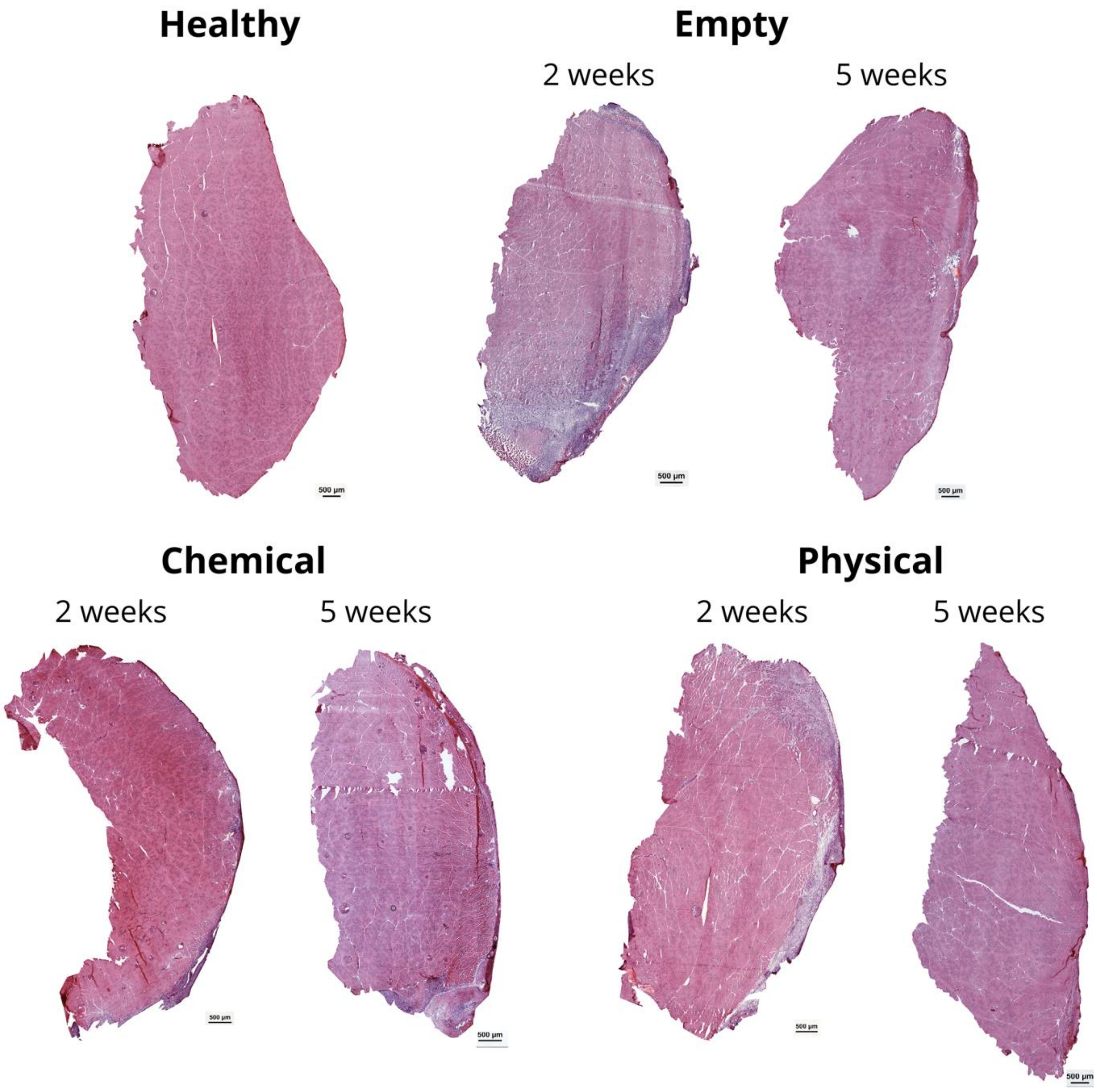

Supplementary Figure 3. Representative images of the hematoxylin-eosin (HE) staining of whole TA muscle cross-sections of samples from the healthy, empty, chemical and physical (right) groups at 2 and 5 weeks post-injury. Scale bar = 500 μm.

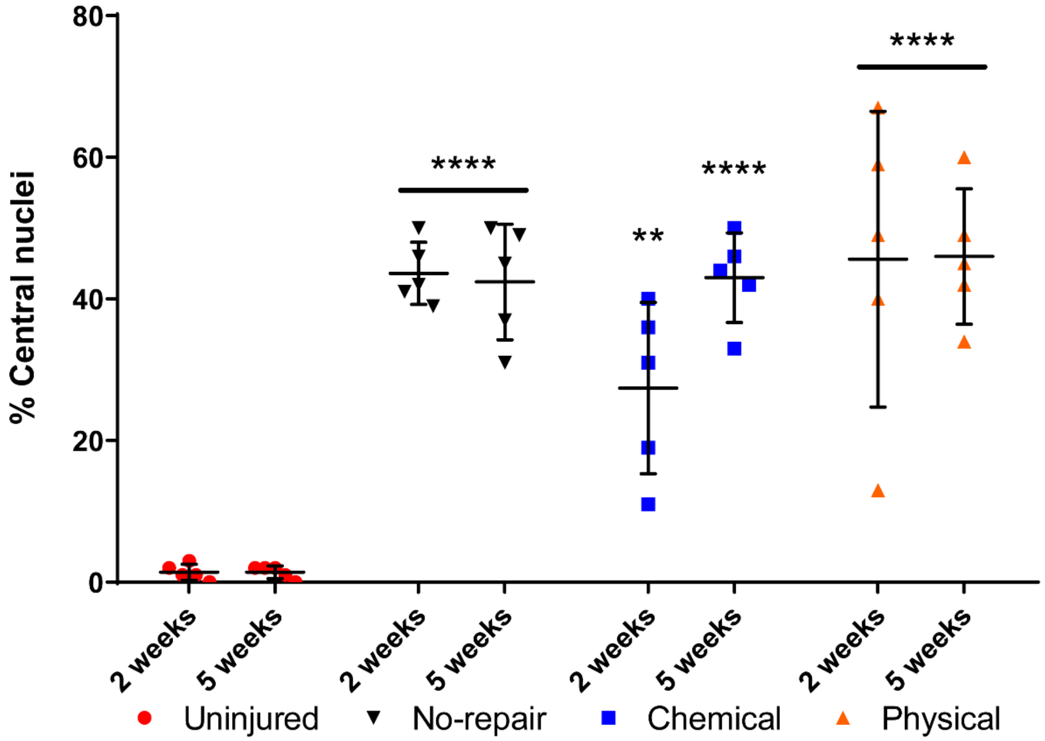

Supplementary Figure 4. Percentage of myofibers with internal nuclei in the interface area for the different groups (uninjured or healthy, red circles; no repair, black inverted triangles; chemical, blue squares, and physical, orange triangles) at 2 and 5 weeks post-injury. The significant differences are shown for the comparison of each experimental group with the uninjured one at each time point (** $p < 0.01$ and **** $p < 0.0001$).

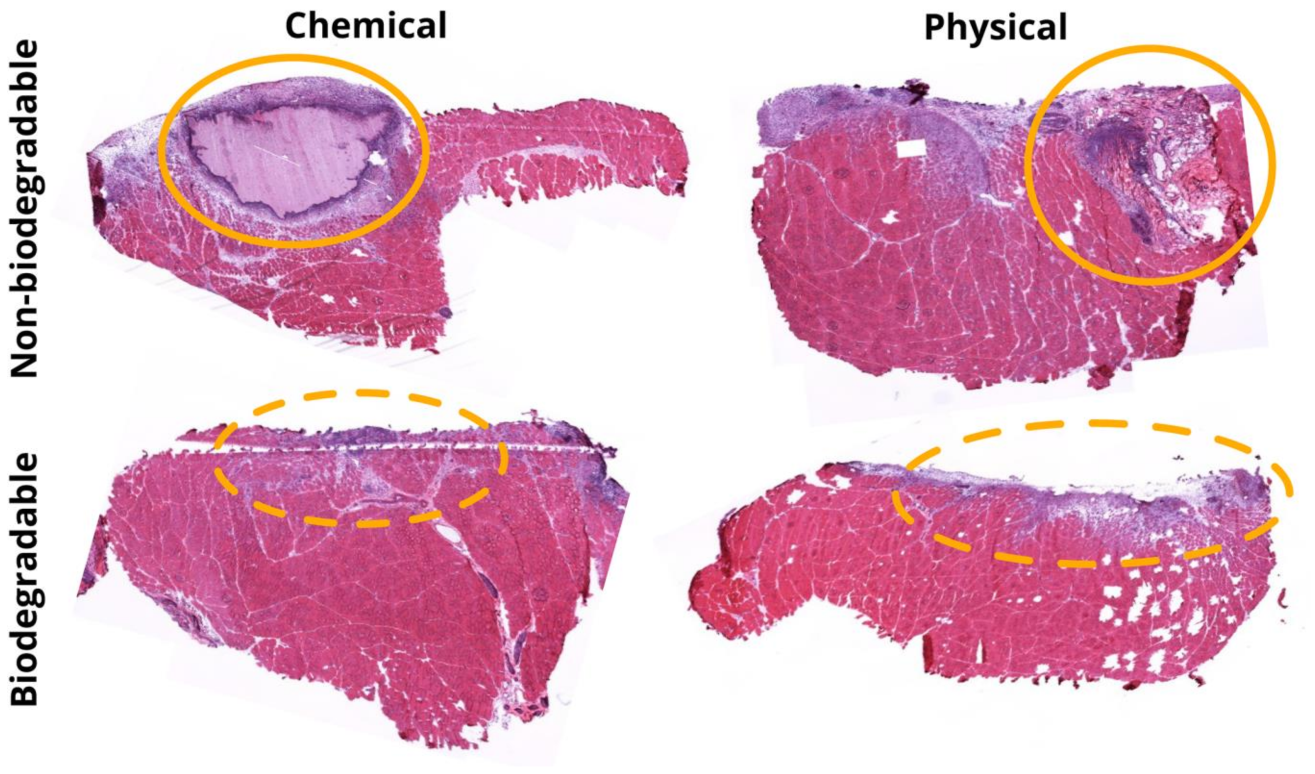

Supplementary Figure 5. Representative images of the hematoxylin-eosin (HE) staining of whole TA muscle cross-sections of samples from the chemical (left) and physical (right) groups, including non-biodegradable (top) and biodegradable (bottom) ELR hydrogels for each type of cross-linking at 2 weeks post-injury. The regions where the hydrogels can be found are highlighted with a complete circumference for the non-biodegradable hydrogels, where the scaffolds can be clearly observed after 2 weeks, and with a dashed circumference for the biodegradable hydrogels, where the scaffolds have been replaced by newly formed skeletal muscle tissue.
